# Mechanisms of Endogenous Neuroprotective Effects of Astrocytes in Brain Injury

**DOI:** 10.1155/2018/6501031

**Published:** 2018-04-01

**Authors:** Michelle A. Bylicky, Gregory P. Mueller, Regina M. Day

**Affiliations:** ^1^Department of Anatomy, Physiology, and Genetics, The Uniformed Services University of the Health Sciences, 4301 Jones Bridge Road, Bethesda, MD 20814, USA; ^2^Department of Pharmacology and Molecular Therapeutics, The Uniformed Services University of the Health Sciences, 4301 Jones Bridge Road, Bethesda, MD 20814, USA

## Abstract

Astrocytes, once believed to serve only as “glue” for the structural support of neurons, have been demonstrated to serve critical functions for the maintenance and protection of neurons, especially under conditions of acute or chronic injury. There are at least seven distinct mechanisms by which astrocytes protect neurons from damage; these are (1) protection against glutamate toxicity, (2) protection against redox stress, (3) mediation of mitochondrial repair mechanisms, (4) protection against glucose-induced metabolic stress, (5) protection against iron toxicity, (6) modulation of the immune response in the brain, and (7) maintenance of tissue homeostasis in the presence of DNA damage. Astrocytes support these critical functions through specialized responses to stress or toxic conditions. The detoxifying activities of astrocytes are essential for maintenance of the microenvironment surrounding neurons and in whole tissue homeostasis. Improved understanding of the mechanisms by which astrocytes protect the brain could lead to the development of novel targets for the development of neuroprotective strategies.

## 1. Introduction: Brain Injury and Cellular Responses

Mechanisms causing damage to the central nervous system (CNS) are numerous and complex, ranging from those associated with age-related neurodegeneration to the acute mechanisms of traumatic brain injury (TBI), ischemic stroke, and radiation exposure. In all cases, however, astrocytes play a central role in the compensatory responses that nature has designed to protect against the loss of terminally differentiated, nonreplicating neurons.

Like aging, acute injuries can result in a long-term progression of pathogenic changes that alter brain functions for years afterwards [1]. Specifically, following an initial TBI, secondary events can occur that extend both the area of as well as the intensity of the injury. Loss of vascular integrity resulting in a breakdown of the blood brain barrier (BBB) causes exposure of the CNS to exogenous immune cell types, abnormal levels of cytokines, and other cellular mediators and ionic disruption that can lead to a cascade of pathogenesis [2–7]. Loss of BBB integrity is also observed following ischemic stroke, radiation exposure, and in certain neurodegenerative disorders, due to the loss of neurovascular functions [8–11]. Secondary damage due to vascular and metabolic imbalances leads to increased glutamate release and subsequent excitotoxicity, mitochondrial dysfunction, and excessive production of reactive oxygen species (ROS), as well as disruption of glucose metabolism/release, and further alterations of ion concentrations [12–14]. Glutamate is thought to be a central mediator in this constellation of secondary injury events. An increase of extracellular glutamate activates N-methyl-D-aspartate receptors (NMDARs) in neurons, allowing calcium influx [15]. The resulting calcium excitotoxicity affects mitochondrial functions, causing a disruption of energy balance and production of excessive ROS, ultimately causing acute necrotic cell death and/or delayed apoptotic cell death [15–18]. Further damage can occur due to prolonged neuroinflammatory and related immune responses that exacerbate the injury [19, 20] and may underlie long-term pathogenesis.

Although the initiating events of CNS damage may differ, similar patterns of secondary injuries are observed [10, 21, 22]. This implies that understanding of the mechanisms underlying the CNS response to any injury may allow the development of treatments for other diseases or disorders.

**Figure 1:**
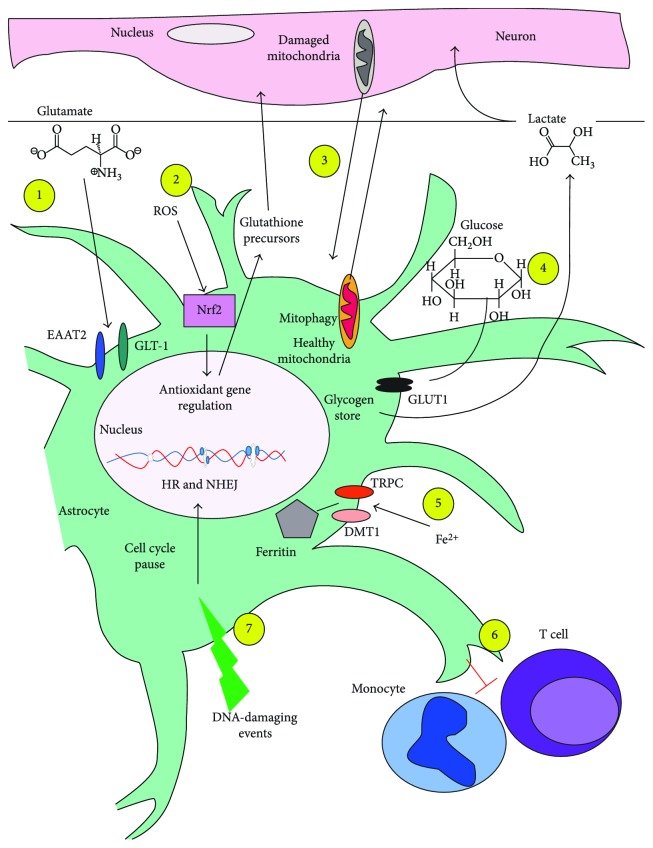
Schematic of mechanisms of neuroprotective effects of astrocytes. There are at least seven distinct mechanisms by which astrocytes protect neurons from damage. (1) Protection against glutamate toxicity occurs through astrocyte uptake of extracellular glutamate through the excitatory amino acid transporter 2 (EAAT2) and the glutamate transporter 1 (GLT-1). (2) Protection against redox stress through the activation of Nrf2 and regulation of antioxidant genes; protection of the neurons is also advanced by the export of glutathione precursors to help neurons synthesize glutathione. (3) Mediation of mitochondrial repair mechanisms by which astrocytes received damaged mitochondria from neurons for mitophagy and in return deliver healthy mitochondria to the neurons. (4) Protection against glucose-induced metabolic stress, which involves astrocytes taking up extracellular glucose for storage as glycogen; the glycogen can be released to neurons as lactate for their metabolism at a later time. (5) Protection against iron toxicity, in which astrocytes sequester free iron for storage in complex with ferritin. (6) Modulation of the immune response in the brain occurs by astrocyte inhibition of both T cell and monocyte activation; the mechanisms for these actions are not fully known. (7) Maintenance of tissue homeostasis in the presence of DNA damage, where astrocytes can effectively repair their DNA through both homologous recombination and nonhomologous end joining, following pause of the cell cycle.

Historically, treatments for acute or chronic damage to the nervous system have focused on neuronal responses and survival. This was due to the neurons' perceived importance in cognition and their postmitotic status which prevents their replacement when damaged [23, 24]. However, more attention is now being paid to the impact of nonneuronal cell types that function to mitigate damage and promote neuronal function and repair following tissue injury. In recent years, there has been a greater appreciation of the role of astrocytes in brain function and survival. The perceived value of astrocytes has risen from their initially defined role of “brain glue” to current findings that astrocytes are critical for modulating synaptic transmissions, managing energy metabolism, water, and ion homeostasis, and protection of neurons from oxidative stress under both mild and catastrophic conditions [25–29]. Here, we review the role of astrocytes in the protection of neurons from the consequences of initial and secondary injury processes (Figure 1).

## 2. Astrocytes: Origin, Morphology, and Activation

Astrocytes are members of a larger family of nonneural, glial cells which include oligodendrocytes and Schwann cells, both of which form myelin and microglia, which are specialized macrophages that aid in immunity. Astrocytes and the other cells of the glial family are defined, in part, by their inability to produce an action potential upon stimulation [30]. Astrocytes are embryonically derived from progenitor cells of neuroepithelium which differentiate to function in their traditional roles as support cells. They provide nutrients and remove end products of metabolism [31]. Astrocytes exhibit spongiform morphology, with processes in close contact with neuronal synapses and other components of the CNS [32]. Recent advances in our understanding of astrocytes, discussed below, reveal the astrocyte to have essential roles in synaptic function and nervous system repair [33, 34].

Astrocytes, the most abundant nonneuronal cell type in the brain, consist of two main subclasses: protoplasmic and fibrous [35]. Protoplasmic astrocytes display a stellate appearance in the grey matter, and fibrous astrocytes primarily exist as long, thin, fibrocyte-like cells in the white matter of the CNS [36]. Each subtype has a distinctive profile of gene expression, as reflected in their expression of specific receptors and proteins [37, 38]. These two types of astrocytes display differences in their development and their expression of receptors and proteins [37, 38]. However, both subtypes express glial fibrillary acidic protein (GFAP), the main astrocytic intermediate filament, as well as calcium-binding S100B protein (S100B) [39, 40].

Activation of astrocytes can occur in response to a variety of injuries to the brain and in response to inflammation or pathological neurodegeneration [35]. The activated state, astrogliosis or reactive astrogliosis, is believed to have multiple functions in the brain and has been the topic of controversy for over 20 years [32, 35, 41]. While in some cases, astrocyte activation has been linked to repair and return to homeostasis, and in other cases, astrocyte activation has been related to the formation of scar tissue and the inhibition of neuronal axon outgrowth [35]. Induction of the reactive state of astrocytes can occur through multiple mechanisms including the presence of amyloid beta peptides (A*β* peptides), to neuronal damage or neurodegeneration, the release of proinflammatory cytokines by microglia and macrophages, or in response to acute injury to cells of the CNS [42–44]. The time course of astrocyte reactivity is heterogeneous and may depend on the location and type of injury [45]. In certain murine models of mild CNS injury, astrocyte reactivity is transient [46]. However, other studies indicate long-lasting increases in astrocyte reactivity occurring after either moderate or severe CNS injury from TBI or by radiation [47, 48]. Mild perturbations of the CNS can be adequately repaired, and homeostasis can be maintained with cooperation among glial cells. However, under more severe conditions, astrocytes remain in a state of reactivity indicating an inability to adequately repair. Similarly, astrocytes in postmortem Alzheimer's patients appear to maintain themselves in a continuous reactive state, consistent with chronic inflammation observed in this disease [49]. Thus, astrocyte reactivity persistence may indicate the presence of unresolved dysfunction in the CNS.

The primary alterations in the transformation of normal astrocytes to reactive astrocytes include hypertrophy of their main cellular processes, proliferation, and alterations in protein expression [32, 50, 51]. Fibrous and protoplasmic astrocytes display differences in the length of their processes following mechanical injury. In a murine model of axonal injury, fibrous astrocytes displayed condensed, retracted processes [46]. In contrast, protoplasmic astrocytes displayed increased length and branch complexity of their processes after injury [32, 52]. This may be a reflection of their functions within the brain, but more research is required to understand the significance of these changes. Of greater interest are their different sensitivities to damage. Research of brain ischemia and cortical lesions has shown that protoplasmic astrocytes may either die or differentiate into fibrous astrocytes after brain injury caused by ischemia and cortical lesions [52, 53]. This suggests that the differences between astrocyte types are fluid and dependent on environmental conditions. Significantly, protoplasmic astrocytes promote the differentiation of neural stem cell (NSC) into neurons via their secretion of brain-derived neurotrophic factor (BDNF) secretion [54]. Also, while both protoplasmic and fibrous astrocytes aid in motor neuron neurite outgrowth, protoplasmic astrocytes produced factors in the extracellular matrix that aided in axonal growth of V2a interneurons, while extracellular matrix produced by fibrous astrocytes had more factors that inhibited axon growth of V2a interneurons, suggesting that the actions of the protoplasmic and fibrous astrocytes are selective for specific neurons [55]. Thus, the differentiation or death of protoplasmic astrocytes may have a significant impact on replenishing neurons and regrowth of neuronal axons in the CNS following injury depending upon the site of injury.

Reactive astrocytes perform a variety of tasks in response to injury which can be beneficial or deleterious to the surrounding neurons, depending on the circumstances of the injury. Reactive astrocytes can form scars after CNS trauma. In some cases, scars can be viewed as initially beneficial since they limit immune cell invasion, decrease neuroinflammation, and maintain ion homeostasis in damaged brain tissue [56, 57]. Ablation of proliferating reactive astrocytes after moderate closed cortical impact (CCI) in mice produced increased inflammation and neuronal death, suggesting that the overall value of astrocyte reactivity is for the protection of neurons postinjury [58]. Evidence indicates interference in the development of the astroglial scar results in increased neuronal cell death and decreased modulation of inflammation [59]. However, there is controversy over its long-term impact of the scar tissue on repair and functional recovery [60, 61]. Prior evidence suggests that glial scar formation prevents or inhibits axonal regrowth of neurons [62]. This has been attributed to astrocyte expression of chondroitin sulfate proteoglycan, a known inhibitor of neuronal axons during embryogenesis [63]. However, in murine models where astrocyte scar formation is impaired, there was demonstrated to be less neuronal axon regrowth and remodeling [64, 65]. Using transgenic murine models, one research group demonstrated that the formation of an astrocytic scar actually improved neuron axonal regrowth, provided that brain-derived neurotrophic factor (BDNF) and neurotrophin-3 (NT3) were added [64]. Together, these studies suggest that, in contrast to initial hypotheses, the presence of astrocytic scars alone does not prevent axonal regrowth, but rather that the lack of adequate growth factors may be the problem.

The beneficial nature of gliosis may become detrimental when damage is too severe for homeostasis to be reestablished. For the purposes of this review, we will focus mostly on the mechanisms by which astrocytes protect neurons under basal conditions and after injury. This will involve focusing on the astrocyte's ability to collect and transport vital nutrients, neurotransmitters, and ions in the brain, to release antioxidants during redox stress, to repair mitochondria and DNA after injury.

## 3. Astrocyte Defense against Glutamate Toxicity

Glutamate is the most abundant excitatory neurotransmitter in the brain, with actions mediated through a diverse family of receptors to modulate synaptic transmission and aid in plasticity [66, 67]. In normal synaptic communication, neurons release measured quanta of glutamate into the synaptic cleft. However, following physical trauma, radiation exposure, and chronic neurodegenerative disorders, including Alzheimer's disease, excessive glutamate is released or fails to be taken up for days after injury [68–71]. The cause of glutamate dysregulation in TBI and neurodegeneration is not completely understood, but elevations in free glutamate are linked to poor clinical outcome [71]. Recent evidence indicates that glutamate is released by dying or damaged neurons, possibly via the cystine glutamate antiporter [72, 73]. Excessive extracellular glutamate leads to excitatory neuronal cell death attributed to overstimulation of NMDAR and subsequent overproduction of ROS in neurons [74, 75].

Under conditions of normal neuronal activity, astrocytes are responsible for the uptake of excess glutamate from the synaptic cleft. Following uptake, astrocytes process the glutamate into glutamine and return it to neurons for reuse [76]. Consistent with this role, astrocytes highly express the excitatory amino acid transporter 2 (EAAT2) and the glutamate transporter 1 (GLT-1) which are responsible for the active uptake of glutamate [77]. Glutamate homeostasis is a critical function of astrocytes in the brain, as demonstrated experimentally by the neurotoxicity that results from inhibition of the astrocyte glutamate transporters [78, 79].

Following tissue injury, astrocytes can actively take up excessive glutamate from the extracellular (nonsynaptic) space and buffer its potential excitotoxic actions on neurons. The reduction of extracellular glutamate by astrocytes decreases the subsequent lesion size, mitigates neuronal death, and improves CNS function postinjury [80].

Under conditions of severe injury, the extent of damage and types of injury to the astrocytes themselves can impact the ability of astrocytes to protect neurons from glutamate toxicity [81]. For example, astrocytes injured by radiation or more severe forms of TBI display reduced glutamate uptake activity as compared to the uninjured condition, allowing increased neuronal uptake of glutamate and a greater extent of neuronal cell death and seizure activity [70, 81, 82]. The mechanism for radiation inhibition of astrocyte uptake of glutamate is thought to be related to ROS inhibition of the astrocytic glutamate transporter via oxidation of protein sulfhydryl groups critical for function [83, 84]. At least three potential mechanisms have been proposed for increased extracellular glutamate levels and subsequent excitotoxicity in TBI [85, 86]. These mechanisms may occur in tandem and are not exclusive. In the first potential mechanism, tumor necrosis factor-*α* (TNF-*α*), the proinflammatory factor released during brain damage, downregulates glutamate uptake by astrocytes and suppresses conversion of glutamate to glutamine [87]. In the second possible mechanism, TBI- and ischemia-induced efflux of glutamate from injured astrocytes may occur in response to thrombin, which is released after BBB disruption [88]. In a third potential mechanism, ischemia and glucose deprivation may induce altered glutamate release by astrocytes [89]. Under normal circumstances, glutamate uptake occurs against its gradient and must be actively transported into the astrocyte via EAATs. However, under acidic conditions, as occurs with hypoxia, this transporter is reversed and expels glutamate [89]. Thus, more severe neuronal injuries and/or chronic disruptions lead to cell death when the astrocytes themselves exacerbate glutamate imbalance as they fail to maintain homeostasis.

## 4. Redox Stress Reduction by Astrocytes

Basal levels of ROS in the brain can result from normal cellular functions and metabolic activity. While the production of ROS is a natural consequence of mitochondrial respiration, overproduction of ROS following injury exceeds the capacity of natural cellular antioxidant mechanisms, resulting in the pathological modification of proteins, lipids, and nucleic acids [90–93]. To combat these processes, the brain utilizes multiple pathways for antioxidant defense including superoxide dismutase (SOD), catalase and glutathione detoxification pathways, and thioredoxin detoxification pathways [94]. These mechanisms are utilized to different degrees by different cell types.

A hallmark of glutamate excitotoxicity is increased intracellular redox stress. Excessive glutamate activation of NMDAR causes Ca^2+^ influx into the cytosol of neurons [95]. The excessive intracellular Ca^2+^ can translocate into the mitochondrial matrix where it leads to the collapse of mitochondrial membrane potential with loss of ATP production and, ultimately, cell death [22, 74]. To prevent this, many cell types upregulate uncoupling proteins (UCPs), which aid in removal of intracellular Ca^2+^ and prevention of Ca^2+^ entry into the mitochondria [96, 97]. UCPs decrease the levels of hydrogen protons in the mitochondrial intermembrane space and therefore the mitochondrial electrochemical proton gradient, by leaking them into the mitochondrial matrix [98, 99]. Since the electrochemical proton gradient is necessary for ATP synthase function, a decrease in hydrogen protons decreases ATP production [100]. The increase of hydrogen protons in the mitochondrial matrix also causes diminished entry of positively charged molecular calcium [101]. In the short term, the activity of UCP may benefit the neurons for immediate survival, but in the long term, it is detrimental, since this process inhibits ATP production [102, 103]. Catastrophic calcium entry due to acute or chronic brain injury can overcome the UCP system, leading to the production of ROS which causes further mitochondrial dysfunction and cell death [22, 104–106]. This mitochondrial membrane depolarization and increase in ROS induced by high Ca^2+^ levels can cause apoptosis by facilitating the release of cytochrome C through the mitochondrial transition pore and activation of caspase 3 [107, 108].

Astrocytes normally display a higher basal level of glutathione (0.91 ± 0.08 mM) as compared to neurons (0.21 ± 0.02 mM), suggesting that under normal conditions, they are capable of detoxification of higher amounts of reactive oxygen and nitrogen species [109, 110]. Astrocytes also have a greater inducible expression of glutathione in response to oxidative stress [111, 112]. The ROS-inducible transcription factor nuclear factor E2-related factor 2 (Nrf2) regulates the glutathione system, as well as the thioredoxin system and SOD [113–115]. Under basal conditions, Nrf2 is constitutively produced and ubiquitinated for degradation by binding to the Kelch-like ECH-associated protein 1 (Keap1) in the cytoplasm [116]. Under conditions of increased oxidative stress, Keap1 binding to Nrf2 is inhibited [117], allowing Nrf2 to escape degradation and instead to translocate to the nucleus where it interacts with the antioxidant response element (ARE) in gene promoters that activate the expression of oxidative stress response genes. Previous research indicated that astrocytes display higher basal and stimulated levels of ARE binding by NRF2 as compared to neurons [118].

Interestingly, Nrf2-induced expression and downstream upregulation of antioxidant defenses in astrocytes confer enhanced resistance to oxidative stress for both astrocytes and neurons [119, 120]. As stated above, the enhanced Nrf2 within astrocytes effectively upregulates antioxidant genes for the protection of the astrocytes [121]. However, Nrf2 expression in astrocytes was also demonstrated to increase neuronal survival in a murine model of amyotrophic lateral sclerosis (ALS) and in vitro in acute hydrogen peroxide exposure [122, 123]. The mechanism by which Nrf2 upregulation in astrocytes allows protection of neurons is complex, and further research is required for a full understanding. However, two mechanisms have been proposed for astrocyte protection of neurons in response to ROS. In the first mechanism, Nrf2 induces glutathione secretion from astrocytes into the extracellular matrix where it is cleaved to one of its precursors (CysGly, *γ*GluCys, or cysteine) which are then taken up and used by neurons for glutathione resynthesis for their own detoxification [21, 124, 125]. In the second mechanism, the increased levels of Nrf2 induce the upregulation of the EAAT3 in astrocytes. As described above, this neurotransmitter transporter is critical for the removal of extracellular glutamate which after injury can induce neuronal excitotoxicity. Thus, the removal of extracellular glutamate protects neurons via a second independent mechanism [126]. This redox buffering capacity of astrocytes was demonstrated to be necessary for neuronal homeostasis under normal basal conditions [127].

## 5. Astrocyte Defense against Mitochondrial Dysfunction in Neurons

As describe in Section 4, brain injury can lead to Ca^2+^-induced mitochondrial dysfunction, including overproduction of ROS, loss of mitochondrial membrane potential and pH gradient, and failure to generate required amounts of ATP [128]. Recently, the transfer of mitochondria from one cell type to another has been described as a mechanism for the replacement and repair of damaged mitochondria. The benefits of mitochondrial transfer were initially shown in cell culture studies in which human mesenchymal stem cells (hMSC) repaired the aerobic respiration of A549-transformed lung epithelial cells that contained mutated mitochondria [129]. Mutant A549 cells which received mitochondria from hMSCs displayed improved ATP production, increased lactate uptake, and higher levels of oxygen consumption, a marker of electron transport chain activity [129]. This study provided compelling evidence for mitochondrial transfer and demonstrated the benefits of this activity as an effective means for protecting vulnerable cell types. The mechanisms by which mitochondria and other organelles are trafficked between different cell types are still not well understood. One proposed mechanism for organelle transfer involves the creation of tunneling nanotubes (TNTs) [130, 131]. TNTs are created by a cell after it is subjected to stress and has been demonstrated to occur during neuronal development [130, 132]. Of special interest, neurons are capable of guiding the formation of astrocyte TNTs during periods of high synaptic activity and thus, high energy demand [132]. Transference of healthy mitochondria from astrocytes to neurons in a murine model of stroke was observed *in vivo* [133]. Further, it was noted in this model that astrocytes only transferred healthy mitochondria to damaged neurons in a calcium-dependent manner, suggesting neuronal activity was necessary for transference [133]. Conversely, in a separate model, it was demonstrated that retinal ganglion cells are capable of shedding damaged mitochondria and that the shed mitochondria were shown to be taken up by adjacent astrocytes where they underwent mitophagy [134]. Thus, evidence suggests that mitochondrial transfer provides means to deliver healthy mitochondria to injured neurons and for the elimination of damaged mitochondria involved in the overproduction of ROS.

## 6. Astrocyte Protection against Glucose-Induced Metabolic Stress

The brain is highly metabolically active, utilizing fully 25% of the body's glucose [28]. Accordingly, efficient glucose uptake and distribution throughout the brain is critical for cognition and survival. Disruptions in the delivery of glucose to the brain induce neuronal cell death. Under normal conditions, the BBB acts as a selective barrier to control entry of glucose into the brain; however, this barrier is often disrupted in brain injury [135]. Endothelial cells of the BBB and astrocytes express glucose transporter 1 (GLUT1), a facilitated glucose transporter, to aid in glucose entry into the brain [136]. Astrocytic endfeet encircle endothelial cells of the BBB and mediate the uptake of glucose [137–139]. Once past the BBB, glucose is taken up by all cell types of the CNS. In astrocytes, glucose is converted into glycogen and stored [140]. In times of need, astrocytes mobilize their glycogen to make lactate available for neuronal use. This is especially important when energy demand is high but neuronal glucose supply is low, such as under hypoglycemic conditions [141–143]. While neurons express glucose transporter 3 (GLUT3), a high affinity glucose transporter, they have been shown to prefer lactate as an energy substrate during times of high synaptic activity [144–146]. Glutamate induces the rapid uptake of glucose in to astrocytes. Because extracellular glutamate is released during neurotransmission, this indicates that glutamate-stimulated glycogen production in astrocytes is linked to neuronal activity [147, 148].

Insulin and insulin-like growth factor 1 (IGF-1) increase glycogen storage in astrocytes but fail to impact glucose transport across the astrocyte cell membrane [149]. However, selective ablation of insulin receptors in mouse astrocytes in vivo results in significantly lower cerebral glucose levels [150]. This indicates a central role for astrocytes in monitoring neuronal metabolic activity and maintaining whole brain energy balance in a manner that is responsive to insulin release in the blood, but in a manner that is different from the regulation that occurs in other tissues.

Acute brain damage, including radiation, TBI, and ischemic stroke, can produce sudden damage to the BBB which can lead to a disruption in the supply of glucose as well as imbalances in extracellular ions. Of particular importance in BBB permeability is increased extracellular potassium that must be removed from the extracellular space [151, 152]. The increase in extracellular potassium may be due to multiple factors including direct cellular injury and secondary mechanisms that compromise potassium buffering by astrocytes [153–155]. While glial cells are capable of buffering normal increases in extracellular potassium, they become overwhelmed under conditions of more severe injuries and the potassium overload can cause death of neurons [155, 156]. Both initial disruption of the BBB and the need to maintain ion homeostasis produce a rapid depletion of glucose and metabolic emergency [151, 152, 157]. Hypo- and hyperglycemic conditions both induce greater cell death in neurons than astrocytes [158–160]. Astrocyte survival in hypoglycemic conditions may rely on several factors including glycogen storage within the astrocytes, alternative energy metabolism of fatty acids, and utilization of antioxidant systems to manage increased oxidative stress [161–163]. In vitro research also demonstrates that astrocytes can improve neuronal survival under situations of glucose disruption by upregulating their respective monocarboxylate transporters (MTCs) which transfers lactate from astrocytes to neurons [164, 165].

While astrocytes may increase their release of lactate after TBI, there is some controversy regarding the possible benefit of this release, as neurons appear less capable of taking up the lactate depending on their level of damage [166, 167]. Increased release of lactate by astrocytes may contribute to lactic acidosis which can exacerbate ischemia-induced oxidative stress [168]. High lactate levels in the cerebrospinal fluid (CSF) of TBI patients have been linked worse clinical outcomes, which is blamed on neuronal mitochondrial dysfunctions, neuronal inability to uptake lactate, and subsequent necrosis in the brain [169]. Increased lactate was also seen in patients after they had seizures caused by severe TBI, with astrocytes potentially releasing lactate as an energy source for these overactive neurons [170, 171]. Under normal homeostasis and conditions of mild-to-moderate injury, astrocytes act to maintain neuronal survival by providing energy resources and maintaining the energy balance of the extracellular environment of the brain, but these actions can produce further damage if the CNS is already severely compromised.

## 7. Astrocyte Mitigation of Iron Toxicity

Astrocytes are responsible for the transfer through the BBB of a variety of nutrients required for brain tissue homeostasis, including iron [172]. Iron performs multiple functions within the brain, serving as an essential cofactor in several enzymatic reactions including those involved in the remyelination of neurons after injury [173, 174]. Iron levels are tightly regulated in the brain via specific transport proteins and metabolic pathways, but dysregulation can occur under pathological conditions [175]. Iron deficiency in the brain, due to causes such as dietary insufficiency or anemia, can produce cognitive impairments [176, 177]. However, an excess of iron, due to TBI, hemorrhagic stroke, or neurodegenerative diseases, causes neurotoxicity [175, 177, 178].

When present at high levels, ferrous iron (Fe^2+^) interacts with hydrogen peroxide to generate toxic levels of hydroxyl radicals through the Fenton reaction [179]. Neuronal susceptibility to iron-mediated necrotic, apoptotic, and autophagic cell death is likely due to their inability to effectively combat redox stress [180]. This is in marked contradiction to astrocytes which are highly effective at detoxification of ROS [181, 182, 183]. Excess iron induces lipid peroxidation, protein and DNA oxidation, and cell death in neurons [175, 184]. Disruptions in free iron handling within the CNS have been observed after acute injuries such as TBI as well as in chronic neurodegenerative disorders [185, 186]. Iron and other transition metals within the brain bind to A*β* a peptide that accumulates in Alzheimer's disease, causing greater neuronal death and toxicity than A*β* alone [187, 188]. Similarly, in a murine model, it was demonstrated that TBI results in an increase in iron deposition in the brain starting as early as four hours postinjury and extending for at least three weeks after initial damage [189]. These findings support the proposal that acute deregulation of iron homeostasis may participate in long-lasting pathogenic effects that underly neuronal damage and death [185, 190] with associated cognitive impairment.

Astrocytes utilize several distinctly different mechanisms to directly regulate free iron in the CNS. As discussed above, astrocytes utilize parallel mechanisms including increased expression of Nrf2, glutathione, and catalase to combat redox stress that is likely one of the consequences of excessive free iron [183, 191]. Astrocytes may also protect neurons from iron-induced cell damage under normal and pathological conditions by sequestering free iron through transient receptor potential canonical (TRPC) channels and divalent metal transporter (DMT1), respectively [192, 193]. TRPC channels are best known for their proposed role in calcium influx after activation, though they transport multiple cation types across the cell membrane [194, 195]. In a cell culture model, it was demonstrated that overexpression of TPRC6 can increase basal levels of intracellular iron and iron presence after stimulation, suggesting that iron transfer through TRPC channels may occur under basal conditions [196]. In contrast, DMT1 expression is controlled by proinflammatory cytokines. The proinflammatory cytokine tumor necrosis factor alpha (TNF-*α*), lipopolysaccharide, and interleukin-6 (IL-6) increase DMT1 expression in astrocytes while simultaneously decreasing ferroportin 1 (FPN-1) expression [197, 198]. FPN-1 is an iron efflux transporter so the result of this activity then is to increase total iron uptake and storage in astrocytes after injury. Excess iron in the microenvironment of astrocytes upregulates the expression of ferritin, a rapidly inducible protein which binds and neutralizes ferrous iron, thus preventing its effects on oxidative stress [199]. Ferritin functions by first converting ferrous iron to its less reactive state of ferric iron then nucleating this ferric iron (Fe^3+^) and storing it within ferritin's iron core [200]. Together, the upregulation of iron transporters plus the upregulation of ferritin allows astrocytes to act as iron stores, resulting in reduced free ferrous iron in the microenvironment where it may contribute to neuronal toxicity.

## 8. Modulation and Regulation of Immune Responses in the CNS

Immunological activity in the CNS is relevant for the prevention of pathogenic infection as well as responses to injury such as stroke and TBI when the BBB is compromised [201]. Astrocytes play a complex role in responding to such CNS insults, and their inflammatory status as well as regulation of immune cells is controversial. Astrogliosis is the defensive reaction of astrocytes to trauma, ischemic damage, inflammation, or pathological neurodegeneration [35]. During astrogliosis, astrocytes increase at the site of the lesion, exhibit altered morphology with increased thicknesses of cellular processes, and display changes in gene expression related to altered function [32, 35]. The increase in astrocytes at the site of injury is believed to be due to proliferation of astrocytes adjacent to the lesion and not due to astrocyte migration from neighboring areas of the brain [35]. Astrocytes can be activated to a proinflammatory or anti-inflammatory phenotype with an associated alteration in their secretome [202–205]. The overall “defensive response” of astrocytes following injury is highly complex and has been shown in some studies to exacerbate inflammation while generally, it is found to mitigate it [35].

The proinflammatory activation of astrocytes and their expression of proinflammatory cytokines are dependent upon the nature of the stimulation they receive and their location in the brain [206]. The activation of proinflammatory astrocytes can occur through multiple mechanisms including interactions with microglia, in response to cytokines such as IL-1, IL-6 oncostatin M, leukemia inhibitory factor (LIF), and transforming growth factor-*α* (TGF-*α*), in response to overt physical damage following brain injury, from interaction with A*β* plaques, or as a result of activation of the calcium-dependent phosphatase calcineurin [35, 41, 207]. Under normal circumstances, astrocytes aid in the morphological and physiological development of neurons and synaptogenesis [208]. However, cell culture studies suggest that inappropriate activation or overactivation of astrocytes can induce the production of TNF-*α* and other cytotoxic factors that inhibit neurite growth and synapse formation [209]. Additionally, exposure of astrocytes in cell culture to cytokines, such as interferon-*γ* (IFN-*γ*), can induce their production of nitric oxide which drives the formation of its toxic metabolite, peroxynitrite [210]. In cell culture, this does not harm astrocytes but can lead to mitochondrial dysfunction and eventual cell death in cocultured neurons [211].

Astrocytes can also modulate the immune system to reduce inflammation. Normal human astrocytes were shown to suppress both monocyte and T cell activation in cell cultures [205]. It was found that astrocytes reduced monocyte activation, not by secreting IL-10, but by blocking CD80 induction on the monocytes through an undefined mechanism [205]. Astrocytes can also function in a manner to recruit and direct white blood cells, both leukocytes and monocytes, to an area of injury, while at the same time protecting healthy tissue from inflammatory consequences of white blood cell invasion [56, 212, 213]. Importantly, ablation of activated astrocytes in a murine model of spinal cord injury resulted in greater inflammation, increased neuronal degeneration, and negatively impacted subsequent motor function, suggesting that the activated astrocytes control the extent and location of inflammation following injury [214]. The mechanisms of suppression of inflammation by astrocytes require further investigation.

## 9. Tissue Homeostasis under Conditions of DNA Damage

DNA repair and synthesis are necessary for normal tissue homeostasis. DNA repair in neurons has been demonstrated to occur in a nonuniform and, in some cases, inefficient manner. Due to a decreased antioxidant response, neurons display increased chromosomal and mitochondrial DNA lesions that can result in cell death [215]. As compared to astrocytes, neurons are slower in rejoining DNA double strand breaks following radiation exposure, and they display greater cell death after episodes of DNA damage [216]. Interestingly, DNA damage in neurons can induce the production of cell cycle enzymes, cyclin B, cyclin E, and proliferating cell nuclear antigen (PCNA) [217, 218]. But this cell cycle progression precedes apoptotic cell death rather than survival and proliferation in neurons [219, 220]. Administration of cell cycle inhibitors after TBI was shown to decrease neuronal cell death [221]. The reason for this response could be related to the hypothesis that slower cycling cells repair DNA more efficiently [222]. In contrast, a murine model of stroke indicated that a pause of several days occurred in the cell cycle of astrocytes before they continue to proliferate after exposure to injury [223]. Such differences in cell cycle control may mean the difference between life and death at the cellular level. An ability to repair effectively before allowing for cell proliferation may explain astrocyte survival postinjury [222].

Neurons do display a limited ability to repair DNA in both wild type and 7,8-dihydro-8-oxoguanine glycosylase (OGG1) deficient mice in response to ischemia [224]. OGG1, a DNA glycosylase involved in base excision repair, protects neuronal mitochondrial DNA from oxidative damage under ischemic conditions [224]. However, the effectiveness of this repair has been called into question and may depend on the location of DNA damage within the chromosome of the mitochondria. Studies of DNA damage from radiation in NTERA-2-derived neurons showed that DNA was efficiently repaired for transcribed genes but inefficiently repaired in nontranscribed areas, suggesting that chromosomal organization plays an important role in the effectiveness of DNA repair mechanisms in neurons [225].

In contrast to neurons, astrocytes display robust DNA repair capacities for both nuclear and mitochondrial DNA [226]. In cell culture assays, astrocytes exposed to menadione, an agent which induces oxidative stress, displayed a lower mitochondrial DNA strand break frequency and more efficient DNA repair as compared to all other cell types of the brain [227]. The mechanisms induced to repair DNA in astrocytes are multifaceted and include upregulation of both primary double strand break pathways: nonhomologous end joining and homologous recombination [228, 229]. Accordingly, astrocytes are better able to protect themselves after DNA damage as compared to neurons. To do this, they utilize a hierarchy of mechanisms. Protection of astrocyte DNA enables prevention of mutations and subsequent loss of function or induction of cell transformation and carcinogenesis. This resilience allows astrocytes to respond to and aid in the protection of neurons and other cell types after brain damage.

## 10. Conclusions

Astrocytes are highly involved in the maintenance and protection of the CNS microenvironment under normal and pathophysiological conditions. Brain damage can begin with mechanical damage to cells, as in TBI, or through oxidative stressors, as in radiation or in neurodegenerative diseases. While the cause of the damage differs, the consequences are similar with an unbalance of extracellular nutrients and ions, damage to the BBB, and excessive release of excitatory neurotransmitters. The resulting conditions can damage mitochondria leading to the production of dangerous levels of ROS that will in turn exacerbate DNA damage and increase inflammation, ultimately leading to cell death. Astrocyte maintenance of the ionic and metabolic environment protects neurons through multiple mechanisms. Astrocytes take up and sequester excess neurotransmitters, ions, and metabolic products to restore the homeostatic balance. Astrocytes also take up and process damaged mitochondria from neurons and transfer healthy mitochondria back to injured neurons. Astrocytes are capable of producing a robust antioxidant response to protect themselves and also neurons, through the release of glutathione precursors to neurons. Their role in scar formation allows astrocytes to regulate and contain the immune responses in a manner that controls neuroinflammation. Further understanding of the endogenous protective mechanisms provided by astrocytes may provide new insights that could lead to the development of novel treatment options for the protection of susceptible cells, such as neurons, under conditions of acute injury or pathology.
